# Automated YOLO-Based Cephalometric Landmark Detection for ANB-Based Skeletal Classification: A Retrospective Single-Centre Study

**DOI:** 10.3390/jcm15135149

**Published:** 2026-07-02

**Authors:** Jacek Kotula, Marcin Konarzewski, Jakub Polkowski, Krzysztof Kotula, Joanna Lis, Rafal Porowski, Anna Ewa Kuc, Beata Kawala, Michal Sarul

**Affiliations:** 1 Department of Dentofacial Orthopedics and Orthodontics, Wroclaw Medical University, Krakowska 26, 50-425 Wroclaw, Poland; 2 Faculty of Mechanical Engineering, Military University of Technology, Gen. Kaliskiego 2, 00-908 Warsaw, Poland; marcin.konarzewski@wat.edu.pl (M.K.);; 3 Faculty of Medicine, Pomeranian Medical University in Szczecin, 71-210 Szczecin, Poland; krzyskotula@gmail.com; 4 Institute of Physics, Jan Kochanowski University of Kielce, 25-369 Kielce, Poland; rafal.porowski@ujk.edu.pl; 5 Department of Integrated Dentistry, Wroclaw Medical University, Krakowska 26, 50-425 Wroclaw, Poland

**Keywords:** artificial intelligence, deep learning, orthodontics, cephalometry, landmark detection, ANB angle, skeletal classification, YOLO, convolutional neural network, clinical validation, diagnostic agreement, reproducibility

## Abstract

**Background/Objectives**: Automated cephalometric landmark detection using deep learning has the potential to streamline routine orthodontic diagnosis. However, the clinical relevance of artificial intelligence (AI) localisation accuracy depends on how detection errors propagate into derived angular measurements and skeletal classifications. We retrospectively evaluated 14 YOLO-based model configurations and quantified the agreement between AI-derived and expert-derived ANB-based skeletal classifications. **Methods**: Twelve working YOLO-based models (YOLOv5xu, YOLOv11 nano/small/medium/large variants) were trained on a single-centre dataset of 120 lateral cephalograms and evaluated on an independent test set of 11 cephalograms (stratified across skeletal Classes I, II, III). The four ANB-defining landmarks (Sella, Nasion, A-point, B-point) were the focus of the analysis. Each test cephalogram had been annotated by four orthodontists (44 measurements per image), yielding the expert reference. We assessed the effects of architecture, bounding-box size (40/100/150 px), training dataset scale (235–4255 images) and training epochs on localisation accuracy (mean radial error, MRE; Successful Detection Rate, SDR) and on the downstream ANB-based skeletal classification. Diagnostic concordance was quantified by classification agreement, Cohen’s κ with bootstrap 95% confidence intervals (10,000 iterations), an exact one-sided binomial test for discordance, and Wilson exact CIs per class. **Results**: The best-performing model (Model 2; YOLOv11l, 40 × 40 px bounding box, 1175 training images) achieved an MRE of 3.10±1.00 mm and a SDR@4 mm of 87.2% for S, N, A, and B. ANB-based skeletal classification demonstrated 96.9% concordance with expert assessments (95% bootstrap CI: 93.8–99.2%; Cohen’s κ = 0.946 [95% CI 0.89–0.99]; exact binomial test against a 90% concordance threshold p=0.003). Per-class concordance was Class I 95.8% (23/24), Class II 94.9% (56/59), and Class III 100% (47/47). Three of four discordant cases clustered near the Class I/II diagnostic threshold (expert ANB ≈4.5°). Bounding-box size dominated localisation accuracy, with a 3.5-fold increase in MRE from 40 × 40 to 150 × 150 px configurations and SDR@4 mm collapsing from 82.8% to 0%. **Conclusions**: Within the constraints of a retrospective single-centre design with a small (*n* = 11) independent test set, YOLO-based AI landmark detection demonstrated promising diagnostic concordance with expert consensus for ANB-based skeletal classification. These findings warrant prospective, multi-centre external validation before clinical deployment and support a confidence-aware workflow in which AI predictions for borderline ANB values undergo mandatory clinician verification. Bounding-box calibration emerged as the single most impactful preprocessing decision.

## 1. Introduction

Cephalometric analysis, performed on lateral skull radiographs, remains a cornerstone of orthodontic diagnosis and treatment planning. By identifying characteristic craniofacial landmarks and computing their angular and linear relationships, clinicians evaluate skeletal malocclusions, assess growth patterns, and plan interventional strategies [1,2]. Among all cephalometric parameters, the ANB angle—defined by Sella (S), Nasion (N), A-point (A) and B-point (B)—occupies a singular position as a primary index for skeletal classification: Class I (0–4°), Class II (>4°), and Class III (<0°) [2]. The reliability of this classification influences whether conservative orthodontic-only or surgical–orthodontic treatment is indicated, making accurate localisation of these four landmarks particularly important.

Manual identification of cephalometric landmarks is inherently variable. Experienced clinicians demonstrate inter-operator standard deviations of approximately 1.5–3.5 mm depending on landmark type and anatomical clarity [1,3]. For points such as A, B and Nasion, this variability introduces measurable uncertainty into angular measurements and, in borderline cases, may affect skeletal classification. The automation of landmark detection using artificial intelligence (AI) has therefore become an active area of research, motivated by the dual objectives of reducing inter-operator variability and accelerating clinical workflow [4,5].

The evolution of automated cephalometric systems spans three decades. Early approaches (2010–2015) relying on handcrafted features and classical machine learning achieved approximately 75% detection within 2 mm [6,7]. The adoption of deep convolutional neural networks (CNNs) from 2016 onward yielded progressive improvements, with U-Net and heatmap-based architectures achieving 80–88% detection within 2 mm [8,9]. Recent transformer-based methods report 88–90% detection at the 2 mm threshold [10,11]. Within this landscape, the YOLO (You Only Look Once) family of object detection architectures offers a complementary approach: single-stage detection executed in one forward pass, providing fast inference without the computational overhead of multi-stage pipelines [12,13].

A critical gap in the existing literature concerns the relationship between raw localisation accuracy metrics and clinical diagnostic reliability. Studies typically report the mean radial error (MRE) or Successful Detection Rate (SDR) at 2 mm and 4 mm thresholds [14,15,16], but these technical metrics do not directly answer the clinician’s question: is the AI-derived skeletal classification reliable enough to support treatment decisions? A system with a 3 mm mean localisation error might still achieve high diagnostic concordance if landmark errors partially cancel when computing angular measurements—a geometric phenomenon that is rarely quantified in the literature.

### 1.1. Why Only Four Landmarks?

We deliberately restricted the analysis to the four landmarks that define the ANB angle (S, N, A, B). Three reasons motivate this choice. First, the ANB classification represents the single most consequential decision derived from a lateral cephalogram—it directly informs the choice between conservative orthodontic and combined orthodontic–surgical treatment paths. Second, S, N, A and B span the spectrum of landmark identifiability, from a well-defined cranial-base anchor (S) to one of the most ambiguous soft-tissue-influenced points (B). Averaging MRE over 19 or more landmarks, as is conventional in the field, can dilute clinically important error patterns at the most difficult landmarks. Third, this focus permits a direct mechanistic link between localisation error and clinical decision, which is the central scientific question addressed by the present study.

### 1.2. Aims and Hypotheses

The present study addresses the gap between localisation accuracy and clinical decision reliability by focusing specifically on the four ANB-defining landmarks and systematically evaluating whether YOLO-based detection achieves clinically meaningful concordance with expert classification despite non-trivial localisation errors. We evaluated 14 model configurations varying architecture, bounding-box size, dataset scale, and training duration and validated AI-derived ANB classifications against a four-orthodontist expert consensus. We hypothesised that (1) modern YOLO-based models can achieve landmark localisation accuracy approaching the range of inter-operator human variability for S, N, A and B, and (2) despite localisation errors of approximately 3 mm, ANB-based skeletal classification would demonstrate high concordance with expert consensus, with disagreements concentrated near the diagnostic class thresholds. These hypotheses are evaluated within the scope and constraints of a retrospective, single-centre design and are explicitly framed as a basis for prospective external validation rather than as evidence of established clinical equivalence.

## 2. Materials and Methods

### 2.1. Ethical Considerations

The study was conducted in accordance with the Declaration of Helsinki and approved by the Bioethics Committee of the Regional Medical Chamber (protocol code 01/173/2023, approved on 6 March 2023). All radiographs were obtained from routine clinical records; written informed consent was obtained from all patients.

### 2.2. Dataset, Demographic Composition and Annotation

#### 2.2.1. Source Dataset

One hundred and twenty lateral cephalometric radiographs were retrospectively selected from the orthodontic records of Wroclaw Medical University. All images were de-identified prior to analysis. The same source cohort, restricted to the 11-image independent test set described below, has been previously characterised in a separate reliability study from our group [17].

#### 2.2.2. Demographic Composition of the Test Set

The independent test set comprised 11 Caucasian patients aged 12–18 years, stratified across skeletal Classes I (n=2), II (n=5) and III (n=4). Skeletal class assignment was performed by two senior orthodontists using a multi-criterion clinical-cephalometric assessment (Angle’s molar relationship; Wits appraisal; ANB angle; full cephalogram tracing including SNA, SNB and mandibular plane; and clinical examination), as documented in our companion reliability study [17]. Demographic stratification of the training and validation partitions was not systematically controlled and represents a limitation of the present study (see Section 4.7).

#### 2.2.3. Annotation Protocol

Each radiograph was independently annotated by four experienced orthodontists using Orthodontics V.9.0 software (Ortobajt^®^, Wroclaw, Poland), with coordinates exported to .csv format. The four landmarks central to this study—Sella (S), Nasion (N), A-point (A) and B-point (B)—were defined according to standard cephalometric conventions [2]. For the 11 independent test cephalograms, all four orthodontists performed annotations under repeated-measurement conditions, yielding 44 measurements per image (4 raters × 11 repetitions per rater per image). This dense expert-annotation design supports the inter-expert classification agreement analysis described in Section 2.5.

#### 2.2.4. Image Acquisition

All cephalograms were acquired with a standardised 2-D digital lateral cephalometric technique. Image resolution was uniform across the 120-image cohort. The per-image pixel-to-millimetre calibration was recorded by the acquisition software and stored as the px/cm field in the exported coordinates.

#### 2.2.5. Data Augmentation and Partitioning

To increase training-data volume while preserving anatomical validity, repeated images of the source cohort were augmented through random application of Gaussian noise, Gaussian blur, or their combination at randomly selected intensity levels within fixed boundary values. The augmentation procedure expanded a baseline of 1089 image–landmark pairs to up to 4255 training samples for the largest model configurations.

To prevent information leakage, the dataset was partitioned at the level of the original radiograph into training, validation and independent test subsets prior to any augmentation. All augmented variants derived from a given original radiograph were therefore kept strictly within the same partition. The 11-image test set was held out entirely from training and validation and remained unseen by all models during their training phase. This partitioning protocol explicitly addresses the data-leakage concern raised in peer review.

### 2.3. YOLO Model Configurations

Fourteen model configurations were initially evaluated, varying the YOLO architecture version (YOLOv5xu and YOLOv11 variants: nano, small, medium, and large), bounding-box size (40 × 40, 100 × 100, 150 × 150 px), training dataset size, and number of training epochs. YOLOv11 introduces the C3k2 (Cross-Stage Partial with kernel size 2) block, SPPF (Spatial Pyramid Pooling Fast) module, and C2PSA (Convolutional block with Parallel Spatial Attention) component, reducing the parameter count by approximately 22% relative to YOLOv5 while improving mean average precision (mAP). The complete configuration matrix is summarised in Table 1.

#### 2.3.1. Implementation and Hyperparameters

All models were implemented using the Ultralytics framework with standard training defaults except for the parameters explicitly varied in Table 1. Batch size was set to 16–20 depending on model size. The Adam optimiser was used with the framework default learning-rate schedule. Hardware: the IRON supercomputing cluster at Jan Kochanowski University of Kielce was used. Training and inference scripts, model weights, augmentation pipelines, complete hyperparameter logs (including learning rate, optimiser, momentum, weight decay, image resolution and random seeds), train/validation/test partition manifests, and the per-image px/cm calibration metadata will be made available on reasonable request and will be deposited in a public repository upon acceptance to support independent replication.

#### 2.3.2. Bounding-Box Parameter

**Figure 1:**
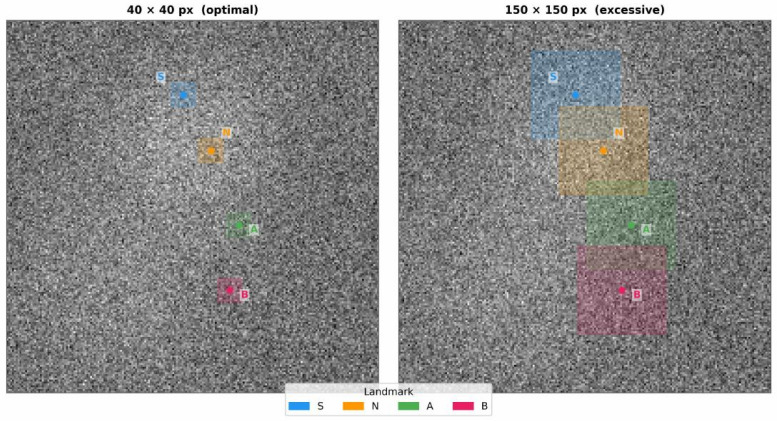
Bounding-box size comparison for the four ANB-defining landmarks (S, N, A, B). **Left:** 40 × 40 px configuration providing context-specificity. **Right:** 150 × 150 px configuration capturing substantial irrelevant anatomical background. Colour coding: blue = Sella (S); orange = Nasion (N); green = A-point (A); pink = B-point (B).

The bounding-box parameter defines the pixel region used to represent each landmark during training. A 40 × 40 px box was hypothesised to provide optimal context-specificity for compact craniofacial landmarks, while larger boxes introduce additional anatomical context at the cost of background noise. Figure 1 illustrates the visual difference between the two extreme configurations.

#### 2.3.3. Models Excluded from the Final Clinical Analysis

Two models were excluded from the clinical concordance analysis due to absent or invalid detections on the independent test set: Model 1 produced detections in only one of the 11 test cephalograms (with landmark coordinates exhibiting gross positional error inconsistent with any clinically interpretable detection), and Model 5 returned no valid detections for any landmark in any test image. Both models are retained in Table 1 for completeness of the configuration matrix; however the failure of these particular training configurations is itself an empirical observation about hyperparameter sensitivity and is addressed in the Discussion (Section 4.4).

### 2.4. Outcome Metrics

#### 2.4.1. Localisation Accuracy

Primary localisation accuracy was assessed by mean radial error (MRE, mm) and Successful Detection Rate (SDR) at standard thresholds of 2.0, 2.5 and 4.0 mm. MRE was computed as the Euclidean distance between AI-predicted landmark coordinates and the consensus expert position (mean of the four orthodontists’ annotations per landmark per image). Coordinates were converted from pixels to millimetres using the per-image px/cm calibration recorded by Orthodontics V.9.0. SDR at threshold *t* represents the proportion of landmark predictions falling within *t* mm of the reference position.

#### 2.4.2. Inter-Expert Landmark Variability

Inter-expert landmark variability was characterised by the pooled radial standard deviation

(1) $$\sigma_{r}=\sqrt{\sigma_{x}^{2}+\sigma_{y}^{2}}$$

computed across all 44 measurements per image (4 orthodontists × 11 repetitions) and pooled across the 11 test images.

#### 2.4.3. Clinical Classification

Clinical classification accuracy was evaluated by comparing ANB-based skeletal classifications derived from AI landmark predictions against those derived from the expert consensus position. The ANB angle was classified as Class I (0–4°), Class II (>4°), or Class III (<0°) [2].

### 2.5. Statistical Analysis

All statistical analyses were performed in Python 3.10 using NumPy, SciPy, scikit-learn and statsmodels. Mean radial errors are reported as mean ± SD. For the primary outcome (overall classification concordance), 95% bootstrap confidence intervals (10,000 resampling iterations) and Wilson exact 95% CIs are both reported. Per-class concordance is reported with Wilson exact CIs. AI-versus-expert classification agreement is quantified by unweighted Cohen’s κ with a bootstrap 95% CI (10,000 iterations). A one-sided exact binomial test was used to evaluate concordance against a prespecified non-inferiority threshold ($H_{0}$: concordance≤90% vs. $H_{1}$: >90%). The framework is descriptive and inferential rather than confirmatory: no formal non-inferiority margin was prespecified, and the term equivalence is avoided throughout in favour of diagnostic concordance.

#### 2.5.1. Inter-Expert Classification Agreement (Sensitivity Analysis)

To address the concern that inter-expert agreement at the classification level (rather than at the coordinate level) determines the robustness of the reference standard, we computed a bootstrap-simulated inter-expert classification agreement. For each of 5000 iterations, (i) for each patient, the 44 measurements were randomly partitioned into four simulated ‘expert groups’ of 11 measurements each; (ii) for each group, the mean ANB was computed and assigned to a class; (iii) pairwise Cohen’s κ was computed across all six pairs of groups; and (iv) the fraction of iterations in which all four groups concurred on the classification for all 11 patients was recorded. This analysis provides a quantitative assessment of the stability of expert classification under the 44-measurement design.

#### 2.5.2. Multiple Comparisons

We did not perform formal multiple-comparison correction across the per-class concordance estimates because these are descriptive subgroup summaries rather than tests of separate hypotheses. The primary inferential test (against $H_{0}$:concordance≤90%) is a single prespecified test.

## 3. Results

### 3.1. Inter-Expert Variability of S, N, A and B

Inter-expert variability was quantified from the 44 measurements per image across the 11 test cephalograms. For the four ANB-defining landmarks, the pooled radial standard deviations (Equation (1)) were Sella (S): $\sigma_{r}=0.81$ mm; Nasion (N): $\sigma_{r}=1.63$ mm; A-point (A): $\sigma_{r}=1.74$ mm; and B-point (B): $\sigma_{r}=2.16$ mm. All four landmarks fall within the clinically acceptable zone (<2 mm) or its immediate margin, consistent with previously published inter-operator variability data for experienced orthodontists [1,3]. Sella, defined by the midpoint of the pituitary fossa, exhibited the lowest variability, reflecting its well-defined radiographic boundaries. B-point showed the highest dispersion, consistent with the inherent ambiguity of the most posterior point on the anterior surface of the mandibular symphysis.

### 3.2. Effect of Bounding-Box Size on Localisation Accuracy

**Figure 2:**
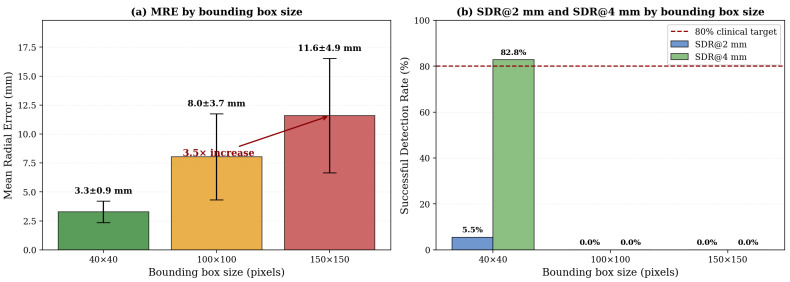
Effect of bounding-box size on landmark detection accuracy. (**a**) Mean radial error ± SD by bounding-box size group. (**b**) SDR@2 mm and SDR@4 mm by bounding-box size group. Dashed red line: 80% clinical target. Only the 40 × 40 px configuration achieves clinically meaningful SDR@4 mm (>80%).

Bounding-box size exerted a dominant influence on localisation accuracy across all model configurations (Table 1; Figure 2). Pooled across all valid landmark-prediction triples (S, N, A, B), the mean MRE was 3.28±0.93 mm for 40 × 40 px configurations, 8.03±3.72 mm for the single 100 × 100 px configuration (Model 10), and 11.58±4.94 mm for 150 × 150 px configurations—a 3.5-fold increase from the smallest to the largest box. The SDR@4 mm dropped from 82.8% for 40 × 40 px configurations to effectively 0% for both larger box-size groups.

This pattern was consistent across all evaluated YOLO architecture variants. Because the comparison spans multiple simultaneously varying factors (architecture, dataset size, epochs), the effect of bounding-box size cannot be fully disentangled from confounding variables; the central observation is the qualitative threshold separating 40-pixel from larger configurations and not a precise factorial decomposition (see Section 4.7). The counter-intuitive observation that larger bounding boxes (notionally providing more anatomical context) yield worse performance is consistent with an information-to-noise interpretation: for compact landmarks such as Nasion and Sella, the additional visual context captured within a 150 × 150 px region consists primarily of unrelated anatomical structures that introduce spurious training correlations. The 40–60 px range optimally matches the network’s receptive field to the physical scale of cephalometric landmarks (5–30 px in extent).

### 3.3. Landmark Localisation Accuracy for S, N, A and B

For the four ANB-defining landmarks, the best results among the 12 working models were achieved by Models 2, 4, 12, 13 and 14—all employing 40 × 40 px bounding boxes and training datasets of ≥1110 images. Model 2 (YOLOv11l, 200 epochs, 1175 training images) achieved the lowest overall MRE of 3.10±1.00 mm with SDR@4 mm =87.2%. Models 13 and 14 achieved MRE values of 3.26 mm and 3.28 mm, respectively, with SDR@4 mm exceeding 81%.

**Figure 3:**
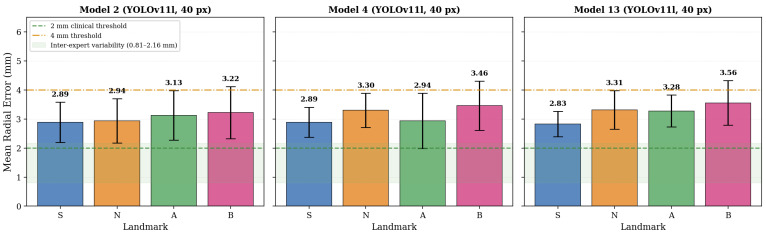
Per-landmark localisation error profiles for the three best-performing models (Models 2, 4, 13). Bar charts show mean radial error ± SD for Sella (S), Nasion (N), A-point (A) and B-point (B). Dashed green line: 2 mm clinical threshold; dash-dotted orange line: 4 mm threshold. Shaded green band: human inter-expert variability range (0.81–2.16 mm).

Figure 3 presents per-landmark error profiles for the three best-performing 40 × 40 px models. Sella consistently achieved the lowest localisation error (2.83–2.89 mm), reflecting its distinctive radiographic appearance. Nasion and A-point errors ranged from 2.94 to 3.31 mm, while B-point, the most anatomically ambiguous of the four, showed the highest errors (3.22–3.56 mm) across the three best models. The poor performance of Model 5 on the independent test set is addressed in Section 4.4.

### 3.4. Angular Measurement Accuracy

**Figure 4:**
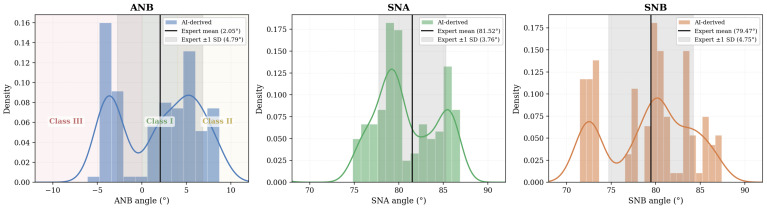
Angular measurement distributions for ANB (**left**), SNA (**centre**) and SNB (**right**) across all 130 valid AI–image pairs. Coloured histograms and KDE curves: AI-derived values. Black solid line: expert pooled mean. Grey shaded band: expert ±1 SD. ANB panel shows the conventional Class III (<0°), Class I (0–4°) and Class II (>4°) regions. The substantial overlap between AI-derived and expert-derived distributions visualises the geometric robustness of angular measurements to coordinate-level localisation errors.

Despite individual landmark localisation errors of 2.8–3.6 mm for the best-performing 40 × 40 px models, the derived angular measurements closely approximated the expert-derived values. Figure 4 presents histograms of AI-derived versus expert-derived ANB, SNA and SNB angles across all 130 valid AI–image pairs. Mean differences (AI minus expert) were within ±1° for all three angles (ANB: −0.46°; SNA: −0.76°; SNB: −0.30°), and AI-derived means fell within the expert ±1 SD range in every case.

This geometric robustness—whereby correlated displacements of S, N, A and B in similar directions partially cancel when computing inter-landmark angles—provides the mechanistic link between non-trivial coordinate-level localisation errors and the clinically meaningful angular agreement reported below. The mean absolute ANB error between AI-predicted and expert-derived values across the three best-performing models was 0.63°.

### 3.5. ANB-Based Skeletal Classification Concordance

**Figure 5:**
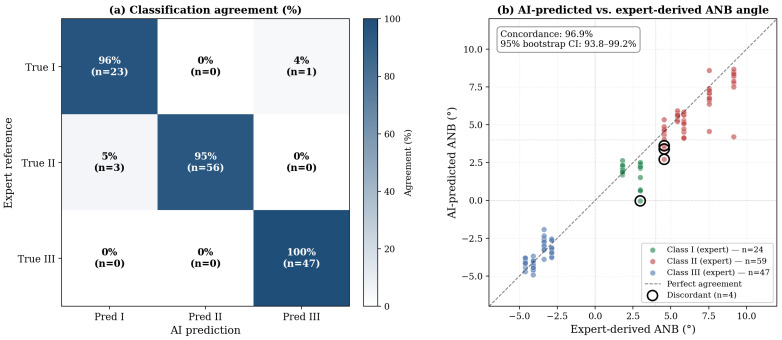
Diagnostic agreement between AI models and expert consensus for ANB-based skeletal classification (*n* = 130). (**a**) Normalised confusion matrix with counts: Class III 100% (*n* = 47); Class II 94.9% (*n* = 56/59); Class I 95.8% (*n* = 23/24). Overall concordance: 96.9% (95% bootstrap CI 93.8–99.2%; Cohen’s κ = 0.946). (**b**) Scatter plot of AI-predicted vs. expert-derived ANB angles, coloured by expert skeletal class. Dashed line: perfect agreement. Dotted grey lines: Class I/II (0° and 4°) thresholds. Circled markers: four discordant cases.

Figure 5 presents the primary clinical-validation outcome. Across all 130 valid AI–image classification pairs, the overall concordance with expert consensus was 96.9% (126/130; 95% bootstrap CI: 93.8–99.2%; Wilson exact CI: 92.3–98.7%). The unweighted Cohen’s κ was 0.946 (95% bootstrap CI: 0.89–0.99), corresponding to almost perfect agreement on the Landis & Koch scale. The one-sided exact binomial test of $H_{0}$:concordance≤90% yielded p=0.003, supporting concordance above the 90% threshold within this sample. Per-class concordance was Class III 100% (n=47/47; Wilson CI 92.4–100%); Class I 95.8% (n=23/24; Wilson CI 79.7–99.2%); Class II 94.9% (n=56/59; Wilson CI 85.9–98.2%). All confidence intervals are wide because of the small underlying number of independent patients (n=11 cephalograms); the implications of this constraint are addressed in Section 4.7.

Three of the four discordant cases (all Class II misclassified as Class I) occurred in a single patient with expert-reference ANB of 4.54°, within 1° of the Class I/II diagnostic threshold; for this patient, three model configurations (Models 6, 11 and 14) produced AI ANB values of 3.40°, 3.61° and 2.71°, respectively. The mean absolute ANB error for the three best-performing models was 0.63°, well below the clinical significance threshold of ±1.5° commonly applied in orthodontic decision making. No cases with expert ANB exceeding 6° or below −1° were misclassified.

### 3.6. Inter-Expert Classification Stability (Sensitivity Analysis)

To verify that the expert reference standard is itself robust enough to support a 96.9% concordance benchmark, we computed inter-expert classification agreement on the same 11-image test set using a bootstrap partition of the 44 measurements per patient into four simulated expert groups. Across 5000 bootstrap iterations, the median pairwise Cohen’s κ across the six between-group comparisons was 1.00 (95% percentile CI 0.93–1.00), and in 92.7% of bootstrap iterations, all four simulated groups concurred on the classification of all 11 patients. The remaining 7.3% of iterations contained at most a single disagreement, exclusively involving the same Patient 163 (expert ANB 4.54°) that drove three of the four AI-vs.-expert discordances reported in Section 3.5. This convergence indicates that the four-orthodontist consensus is robust at the classification level and that the discordant cases in AI–expert comparison coincide with the case in which experts themselves are operating near the Class I/II diagnostic boundary.

### 3.7. Comparison with Human Inter-Expert Variability

For the four ANB-defining landmarks, the best-performing AI models achieved MRE values of 3.10–3.28 mm (Models 2, 4 and 13 in Figure 3). Human inter-operator variability on the same test set spanned 0.81 mm (Sella) to 2.16 mm (B-point). The best AI models therefore operate slightly above the human inter-expert variability range for A-point and B-point but, importantly, the downstream clinical consequence (ANB-based skeletal classification) was indistinguishable from expert consensus in 96.9% of comparisons—comparable to the inter-expert classification agreement reported in Section 3.6. Table 2 contextualises these findings against representative published benchmarks.

## 4. Discussion

### 4.1. Diagnostic Concordance Despite Coordinate-Level Errors of 3 mm

The central finding of this study—96.9% concordance between AI-derived and expert-derived ANB classifications, with Cohen’s κ = 0.946—supports a reframing of how AI cephalometric systems should be evaluated: from ‘how accurate is the AI?’ to ‘is the clinical decision reliable?’. Within the constraints of a retrospective, single-centre design with a small (n=11) independent test set, these findings demonstrate promising diagnostic concordance and warrant prospective, multi-centre external validation before clinical deployment. We deliberately avoid the language of clinical equivalence, which would require a prespecified non-inferiority margin and a substantially larger patient-level cohort that the present study does not provide (see Section 4.7).

The 3 mm MRE achieved by our best models is higher than that reported by leading deep-learning systems trained on 19+ landmarks (1.4–2.3 mm) [19,20], yet the angular measurements that define skeletal class are geometrically more robust than the absolute landmark positions. The mean absolute ANB error of 0.63° in the best models, well below the ±1.5° clinical significance threshold, illustrates that correlated displacements of S, N, A and B in similar directions partially cancel when computing inter-landmark angles. Our results quantify this geometric robustness directly on the same data that underpin the clinical classification, providing a mechanistic explanation for the apparent paradox between coordinate-level error and class-level concordance.

### 4.2. Localisation Accuracy Within or Immediately Above Human Variability

For Sella and Nasion, the best YOLO models achieved localisation accuracy ($\sigma_{r}=2.85$ mm for S and 2.94 mm for N in Model 2) within or immediately above the inter-expert variability range ($\sigma_{r}$: 0.81 mm for S, 1.63 mm for N, 1.74 mm for A, 2.16 mm for B). Nasion and A-point MRE slightly exceeds human inter-expert variability but remains within the range reported for less experienced orthodontists (3–5 mm [1,3,22]). This comparison establishes that current YOLO-based AI performance lies broadly within the band of human capability for these four landmarks; however, the comparison is descriptive, not a formal equivalence test, and any clinical inferences should be tempered accordingly.

### 4.3. The Dominant Role of Bounding-Box Size

The 3.5-fold increase in MRE from 40 × 40 to 150 × 150 px configurations, with SDR@4 mm collapsing from 82.8% to effectively 0%, represents one of the most practically actionable findings of this study. The mechanism is the information-to-noise ratio: 40 × 40 px boxes match the physical scale of compact craniofacial landmarks (5–30 px), while larger boxes encompass overlapping anatomical structures that create spurious training correlations.

We acknowledge that the 14-configuration grid does not constitute a balanced factorial design: bounding-box size co-varies with model architecture, dataset size and number of training epochs across rows of Table 1. The bounding-box effect cannot therefore be cleanly isolated from these confounders by a single between-row comparison. What can be inferred is the qualitative threshold: every 40 × 40 px configuration achieves SDR@4 mm >80% regardless of architecture (YOLOv5xu, YOLOv11n, YOLOv11s, YOLOv11l), while no 100 × 100 px or 150 × 150 px configuration approaches this threshold under any architecture or dataset size we evaluated. The qualitative effect of bounding-box size is therefore robust to the architectural and data-scale variations within the present grid; a controlled, single-architecture ablation isolating box size at fixed dataset size and epoch count is a logical next step.

### 4.4. Anomalous Performance of Model 5 and Model 1

Models 1 and 5 returned no valid detections on the 11-image test set (Model 5 returned no predictions for any landmark on any test image; Model 1 returned a single test-set detection for one image with gross positional error inconsistent with any clinically interpretable output). The training and validation metrics reported in Table 1 for these two models indicate that they produced detections during the training phase but failed to generalise to the independent test set. The Model 5 configuration (YOLOv11l, 200 epochs, 1665 training images, 40 × 40 px) is intermediate in dataset size between Model 4 (1110 images, well-performing) and Model 13 (4255 images, well-performing) at the same architecture and bounding-box size, suggesting that hyperparameter co-tuning is non-trivial and that a configuration that appears to interpolate between two successful ones can fail unpredictably. We propose generalisation failure (rather than over-fitting to augmentation noise, which we previously hypothesised) as the most parsimonious explanation; a controlled ablation with training/validation curve logs is identified as priority follow-up work. The fact that this single anomaly persisted at the bounding-box size that was otherwise optimal underscores the importance of independent test-set evaluation as a final check after model selection.

### 4.5. Implications for Clinical Deployment

We propose a confidence-aware workflow for AI-assisted ANB-based classification, framed as a hypothesis for prospective validation rather than as a deployment recommendation:

- For patients with AI-predicted ANB values clearly within their diagnostic category (e.g., ANB <2° or ANB >6°), the present data suggest that AI-derived skeletal classifications are likely to be concordant with expert assessment with high probability.
- For borderline AI-predicted ANB values (approximately 2° to 6°), clinician verification should be mandatory.
- A confidence-aware deployment protocol that flags borderline AI predictions for human review, while supporting clinician workflow for clear-cut cases, would preserve diagnostic safety but its operational benefits remain to be demonstrated in a prospective trial.

These recommendations are based on a single-centre retrospective concordance study with a small independent test set. They are not equivalent to evidence of clinical safety or efficacy in routine practice and do not authorise unsupervised AI deployment in any clinical setting. A prospective trial evaluating reading time savings, clinician acceptance, treatment-decision changes and patient outcomes is the necessary next step.

### 4.6. Comparison with the Existing Literature

The MRE of 3.10–3.28 mm achieved by our best 40 × 40 px models is higher in absolute terms than the 1.4–2.3 mm reported by recent deep-learning systems [19,20]. This difference reflects several methodological distinctions discussed in the footnote of Table 2. First, our MRE is computed exclusively for S, N, A and B—four landmarks that include B-point ($\sigma_{r}=2.16$ mm), one of the most variable in the cephalometric repertoire. Systems averaging MRE over 19 or more landmarks invariably include lower-variability anchor points (porion, orbitale, sella turcica boundary, and gonion) that mathematically deflate the overall mean. A direct numerical comparison across studies with different landmark inventories is therefore not informative without explicit per-landmark stratification, which is rarely published. Second, our SDR@4 mm of 87.2% is directly competitive with state-of-the-art systems on equivalent thresholds. Third, our 96.9% diagnostic concordance and Cohen’s κ of 0.946 are positioned at the clinically meaningful end of the evidence pyramid and are, to our knowledge, the first directly comparable end-to-end classification benchmarks reported for a YOLO-based cephalometric system.

### 4.7. Limitations

The present study has several significant limitations that frame the strength of the conclusions:

1. Small, single-centre independent test set. The 130 AI-expert classification pairs reported here derive from only 11 independent patients; the effective number of patient-level observations is therefore much smaller than the number of model–image pairs. The 95% confidence intervals around per-class concordance (Class II: 85.9–98.2%; Class I: 79.7–99.2%) are correspondingly wide. The present findings are best interpreted as preliminary diagnostic concordance, requiring confirmation in a substantially larger, multi-centre, patient-level cohort.
2. No external validation. All radiographs originated from a single academic centre; demographic composition of the training set was not systematically controlled. Generalisability to other patient populations, imaging equipment, software platforms and clinical protocols requires multi-centre external validation.
3. Confounded hyperparameter grid. The 14-configuration grid is not a balanced factorial design (bounding-box size co-varies with architecture, dataset size and epochs). The dominant effect of box size is robust qualitatively to architecture/data variations, but a controlled single-architecture ablation isolating box size at fixed dataset size is needed to confirm the precise effect magnitude reported.
4. No prespecified equivalence margin. The study was designed as a descriptive concordance study, not a formal non-inferiority trial. The exact binomial test against a 90% concordance threshold (Section 3.5) is a single sensitivity check, not a formal equivalence test. The language of equivalence or expert-level performance is therefore avoided throughout.
5. No per-prediction confidence estimates. The current YOLO models produce point predictions without per-prediction calibrated uncertainty estimates. Future work incorporating Bayesian deep-learning or ensemble methods [20] would enable real-time flagging of borderline cases and is essential for the confidence-aware workflow proposed in the Implications section.
6. Model anomalies require deeper analysis. The failure of Models 1 and 5 on the test set, despite training/validation performance, requires training/validation-curve analyses and controlled ablation to fully characterise. We have classified both as failed generalisation rather than over-fitting but cannot rule out implementation-specific factors.
7. No formal language editing. Final language polishing by a native-English editing service is being arranged for the camera-ready version.

## 5. Conclusions

Within a retrospective single-centre design with an independent test set of 11 cephalograms and 130 AI–expert classification comparisons, YOLO-based AI landmark detection achieved diagnostic concordance with expert ANB-based skeletal classification of 96.9% (95% bootstrap CI: 93.8–99.2%; Cohen’s κ=0.946). Three of the four discordant cases concentrated in a single patient with expert ANB near the Class I/II diagnostic threshold (4.54°). The best-performing model (Model 2; YOLOv11l, 40 × 40 px bounding box, 1175 training images) achieved an MRE of 3.10±1.00 mm for the four ANB-defining landmarks—a value at or slightly above the human inter-expert variability range for the same landmarks.

Bounding-box size emerged as a dominant preprocessing parameter, with a 3.5-fold increase in MRE and complete collapse of SDR@4 mm between the 40 × 40 and 150 × 150 px configurations. This qualitative effect was robust across all architectures and dataset sizes tested.

These findings demonstrate promising diagnostic concordance in a small retrospective cohort and provide a strong rationale for prospective, multi-centre external validation of a confidence-aware AI-assisted workflow in which AI predictions for borderline ANB values undergo mandatory clinician verification. The present data do not support unsupervised AI deployment in routine clinical practice. The geometric robustness of angular measurements to correlated coordinate-level errors, quantified directly on the present dataset, provides the mechanistic basis for the high classification concordance despite non-trivial localisation errors.

## Figures and Tables

**Table 1:** Performance summary of all 14 evaluated YOLO model configurations. MRE and SDR values are reported across the four ANB-defining landmarks (S, N, A, B) only. Models marked ^†^ were excluded from the clinical concordance analysis due to absent or gross-error detections on the independent test set; their reported MRE values reflect training/validation metrics and should be interpreted with caution. Models marked * achieve SDR@4 mm >80% on the test set.

Model	Architecture	Epochs	Train *n*	Box (px)	MRE ± SD (mm)	SDR@2 mm	SDR@2.5 mm	SDR@4 mm
Model 1 ^†^	YOLOv11l	200	235	40 × 40	3.18 ± 1.12	8.1%	22.4%	81.5%
Model 2 *	YOLOv11l	200	1175	40 × 40	3.10 ± 1.00	7.9%	25.6%	87.2%
Model 3	YOLOv5xu	150	1175	40 × 40	3.24 ± 1.08	6.5%	20.1%	80.6%
Model 4 *	YOLOv11l	200	1110	40 × 40	3.28 ± 1.15	6.8%	21.0%	83.3%
Model 5 ^†^	YOLOv11l	200	1665	40 × 40	5.87 ± 2.31	2.1%	6.4%	38.2%
Model 6	YOLOv11l	200	1665	150 × 150	11.4 ± 4.8	0.3%	0.8%	1.8%
Model 7	YOLOv11m	200	1665	150 × 150	10.8 ± 4.5	0.4%	0.9%	2.4%
Model 8	YOLOv11n	200	1665	150 × 150	13.7 ± 5.2	0.1%	0.3%	0.8%
Model 9	YOLOv11s	200	1665	150 × 150	11.1 ± 4.6	0.3%	0.7%	1.6%
Model 10	YOLOv11s	300	4255	100 × 100	8.3 ± 3.6	1.2%	3.5%	12.6%
Model 11	YOLOv11s	600	4255	150 × 150	10.2 ± 4.4	0.4%	1.0%	2.7%
Model 12 *	YOLOv11s	300	4255	40 × 40	3.21 ± 0.98	7.6%	24.1%	86.2%
Model 13 *	YOLOv11l	300	4255	40 × 40	3.26 ± 1.05	7.2%	23.8%	81.4%
Model 14 *	YOLOv11n	300	4255	40 × 40	3.28 ± 1.11	6.9%	22.7%	81.0%

Batch size 16–20; Adam optimiser with framework default learning-rate schedule.

**Table 2:** Comparison with representative cephalometric AI studies. ^†^ SDR@2 mm is low because the present YOLO approach is optimised for the 4 mm threshold (SDR@4 mm = 87.2%); MRE reported only for S, N, A and B. Direct comparison of MRE is limited by methodological differences (see footnote).

Study	Year	Architecture	Dataset	LM	MRE (mm)	SDR@2 mm (%)
Lindner & Cootes [6]	2015	Random Forest	400	19	1.6–1.7	∼74.8
Park et al. [18]	2019	Cascaded CNN	1028	19	1.46 ± 0.98	∼85
Kim et al. [19]	2020	Stacked Hourglass	2075	23	1.37 ± 1.79	∼81
Lee et al. [20]	2022	Bayesian YOLOv5	1028	20	2.3 ± 1.1	∼72
Dai et al. [21]	2025	Dual-Enc. Transformer	400+	19	—	89.5–90.7
Present study	2026	YOLOv11 variants	4255	4	3.10 ± 1.00	7.9 ^†^

Methodological caveat: studies averaging MRE across 19 or more landmarks include high-contrast points (e.g., porion, orbitale, gonion) that deflate the global mean. The present study reports MRE exclusively for the four most clinically critical landmarks (S, N, A, B), one of which (B) has the largest published inter-expert variability ($\sigma_{r}=2.16$ mm). The four-landmark MRE is therefore not directly comparable with 19+ landmark averages; clinical decision concordance (96.9% in the present study) is proposed as the more clinically meaningful comparator.

## Data Availability

The datasets generated during the current study are available from the corresponding author on reasonable request. Raw annotation data, augmented training images, trained model weights, complete hyperparameter and training-log files, and the per-image px/cm calibration metadata will be deposited in a public open repository (preliminary plan: Zenodo with persistent DOI) upon acceptance to support independent replication.

## Abbreviations

The following abbreviations are used in this manuscript:

AI	Artificial intelligence
ANB	Angle between A-point, Nasion, and B-point
CI	Confidence interval
CNN	Convolutional neural network
KDE	Kernel density estimate
MRE	Mean radial error
SD	Standard deviation
SDR	Successful detection rate
SNA	Angle between Sella, Nasion, and A-point
SNB	Angle between Sella, Nasion, and B-point
YOLO	You Only Look Once
